# In-stent Restenosis in an Anomalous Left Main Coronary Artery Arising from the Right Sinus of Valsalva After a Stenting Lesion with Acute Angle

**DOI:** 10.7759/cureus.7204

**Published:** 2020-03-08

**Authors:** Wail Alkashkari, Alaa Meer, Attafah Omeish, Mohammed Althobaiti, Abdulhalim J Kinsara

**Affiliations:** 1 Cardiology, King Faisal Cardiac Center, King Abdulaziz Medical City, Jeddah, SAU; 2 Cardiology, King Saud Bin Abdulaziz University for Health Science, Jeddah, SAU; 3 Medical Research, King Abdullah International Medical Research Center, Jeddah, SAU; 4 Cardiology, King Faisal Cardiac Center, King Abdulaziz Medical City, Ministry of National Guard Health Affairs, Jeddah, SAU; 5 Radiology, King Abdulaziz Medical City, Ministry of National Guard Health Affairs, King Abdullah International Medical Research Center, King Saud Bin Abdulaziz University for Health Science, Jeddah, SAU; 6 Cardiology, Ministry of National Guard Health Affairs, King Abdullah International Medical Research Center, King Saud Bin Abdulaziz University for Health Sciences, King Abdullah International Research Center, Jeddah, SAU

**Keywords:** restenosis, angioplasty, balloon, coronary, drug-eluting stent

## Abstract

We describe a 38-year-old male who underwent percutaneous coronary intervention (PCI) using a third-generation drug-eluting stent (DES) with a thin stent for an anomalous left main coronary artery (LMCA) originating from the right coronary sinus with a retro-aortic course. Six months later, in-stent restenosis (ISR) occurred due to stent implantation in angled lesions with significant hinge motion. An intravascular ultrasound (IVUS) revealed significant neointimal hyperplasia. The vessel wall of an angled coronary artery lesion is exposed to mechanical stress from the deployed stent. It has been reported before in the major coronary arteries but not in an anomalous LMCA.

## Introduction

An angled coronary lesion, which is often accompanied by hinge motion, is reportedly a risk factor for in-stent restenosis (ISR) with all generations of coronary stents [1]. Several mechanisms are behind the higher incidence of ISR in angled lesions [2]. Initially, it was described after bare-metal stent (BMS) implantation. Drug-eluting stents (DES) have dramatically reduced the rate of ISR as compared with BMS. However, ISR in angled lesions remains a problem, especially with old-generation DES having a closed-cell, stainless-steel design, which has low conformability and flexibility. Recently, third-generation DES, which have thin stent struts and an open-cell design, lead to better conformability and flexibility at the expense of weak radial strength and the risk of stent fracture, especially at an angled lesion. Several studies have reported the relationship between angled lesions with hinge motion and ISR after DES implantation [3]. We describe a case of ISR due to an acute angle lesion with hinge motion after third-generation DES implantation for the first time in an anomalous LMCA.

## Case presentation

**Figure 1:**
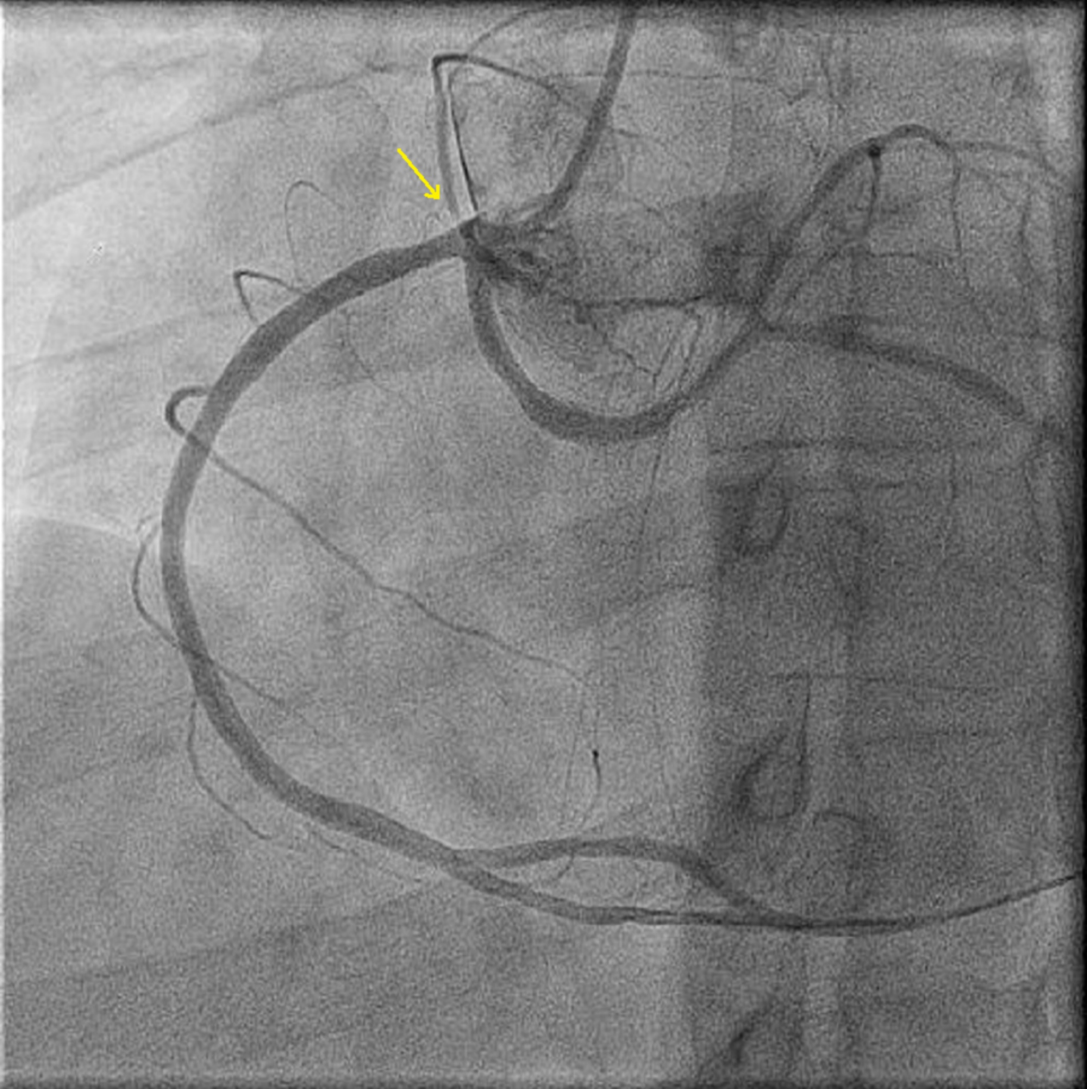
Coronary angiography showing right and left coronary arteries originating from the right coronary sinus of Valsalva

**Figure 2:**
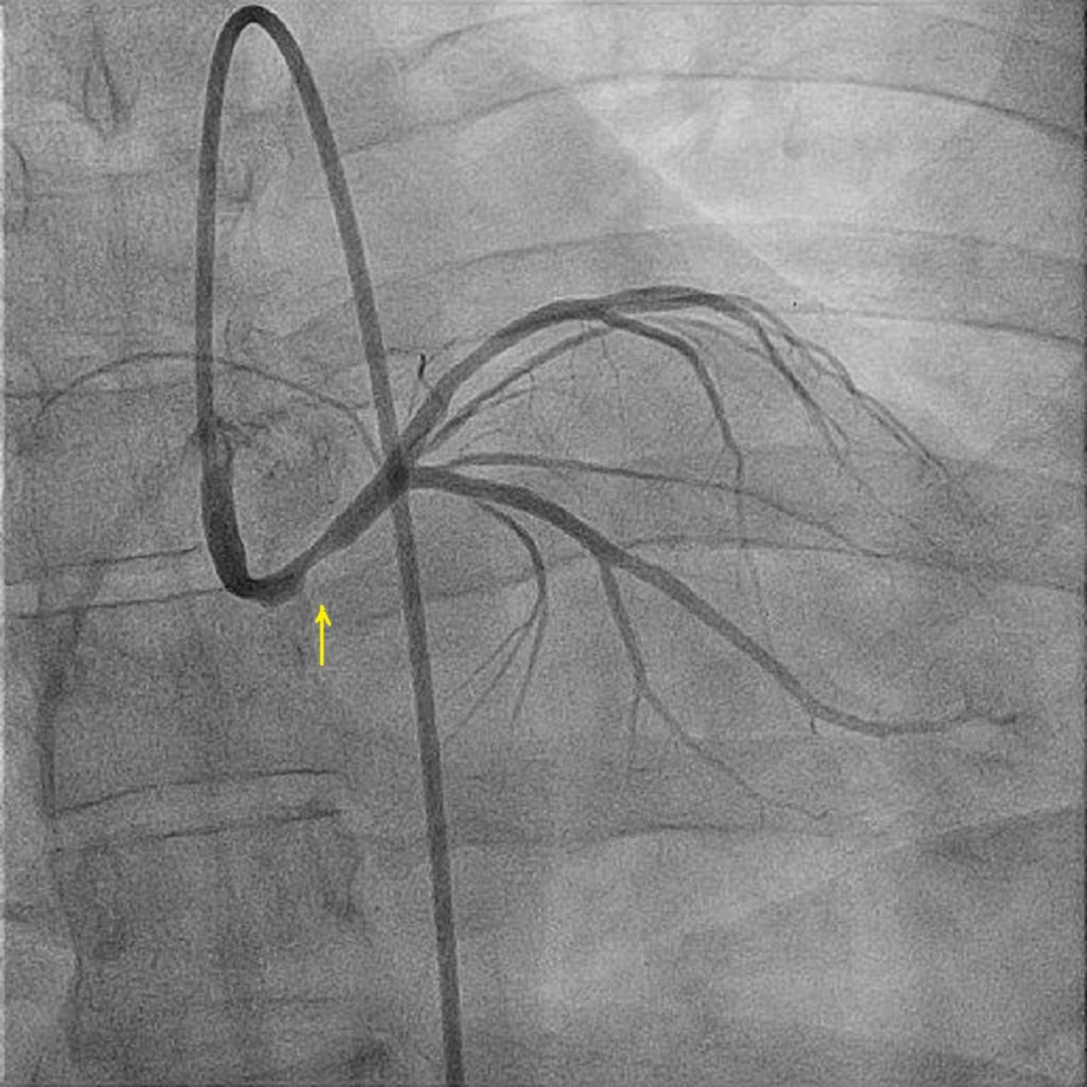
Coronary angiography showing right and left coronary arteries originating from the right coronary sinus of Valsalva

**Figure 3:**
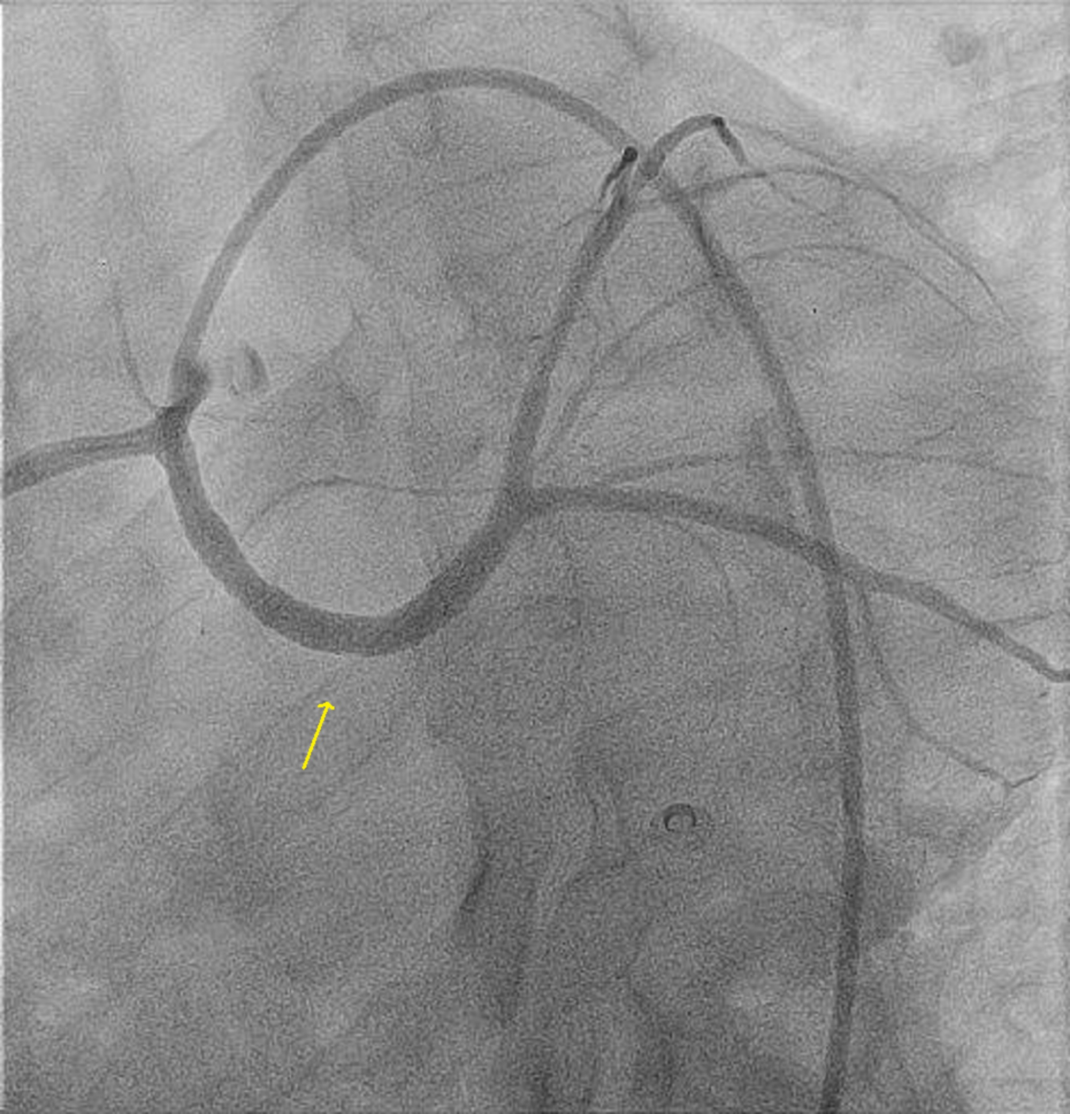
Post stenting of the anomalous left main

**Figure 4:**
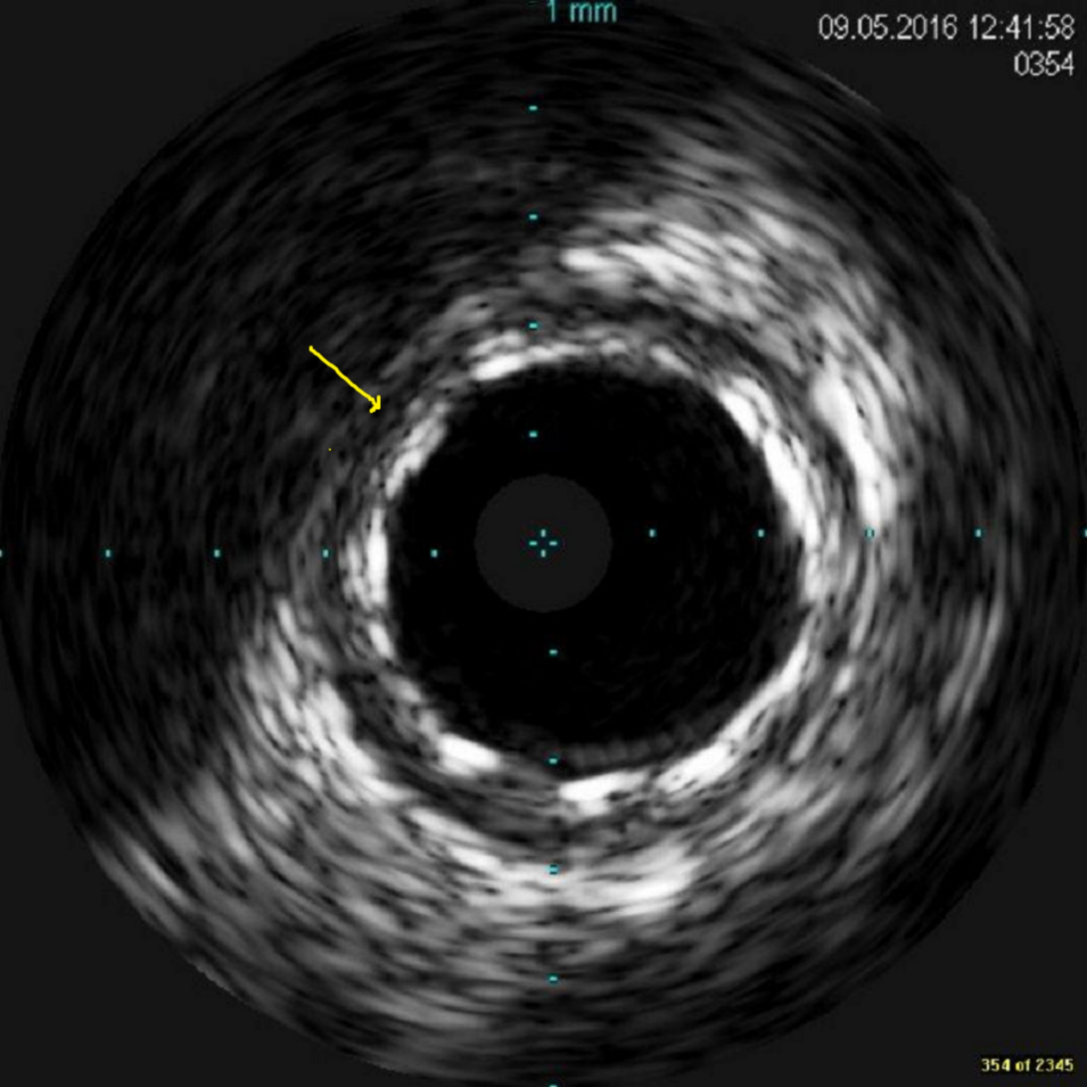
The final intravascular ultrasound image of the stented segment with excellent apposition

A 38-year-old male patient with a 15-pack per year smoking history presented to another hospital with acute anterolateral ST-elevation myocardial infarction. He was thrombolyzed with t-PA with the complete resolution of his electrocardiography (ECG) changes. Echocardiogram revealed mild left ventricular (LV) dysfunction with a left ventricular ejection fraction (LVEF) of 40%-45% and anterior/lateral wall hypokinesis. He was then transferred to our center where he was taken to the catheterization laboratory and a diagnostic coronary angiogram was done after obtaining informed consent. The angiogram revealed a normal right coronary artery and an anomalous origin of the left main coronary artery from the right coronary cusp (Figure 1). The anomalous left main showed a mid-shaft hazy and eccentric lesion (Figure 2). Normal left anterior descending and normal left circumflex were present. The patient was discussed in a heart team meeting and it was decided to proceed with a coronary CT angiogram to define the course of the anomalous LMCA, which was retro-aortic. The patient's informed decision was to opt for percutaneous coronary intervention (PCI). A percutaneous intervention was carried out using a right Judkins 4 guiding catheter and a balance middle weight (BMW) wire that passed to the distal left anterior descending artery (LAD) and a direct 4.0x22 mm resolute stent deployed (Figure 3). A second 4.0x8 mm resolute stent was deployed proximally to cover an area of plaque shift. This was followed by post-stent balloon dilatation using a 4.5X15 mm non-complaint balloon at high pressure. A final intravascular ultrasound (IVUS) was done, which revealed good stent apposition (Figure 4). The patient discharged home within 48 hours [4].

**Figure 5:**
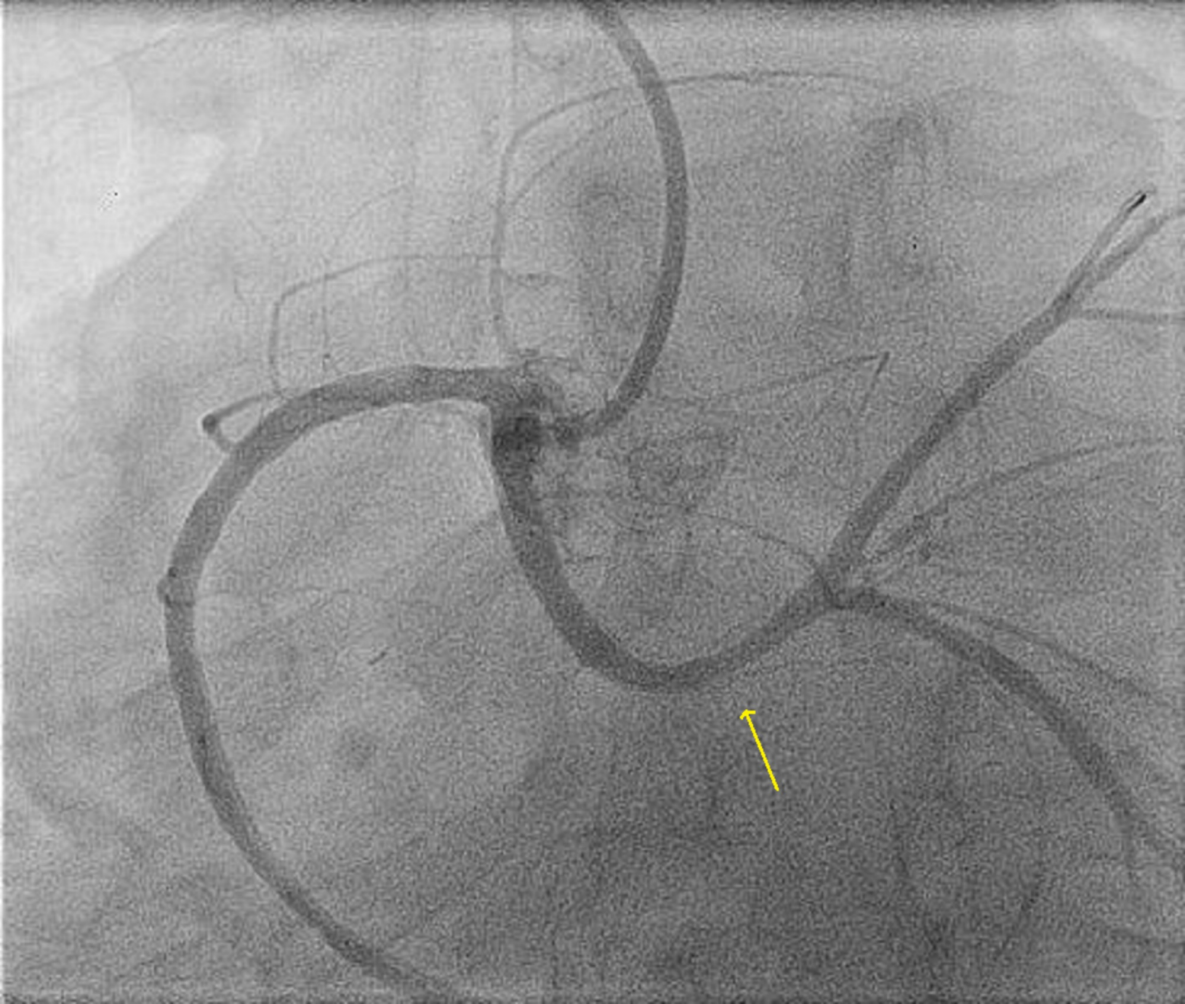
Type three left main in-stent restenosis

**Figure 6:**
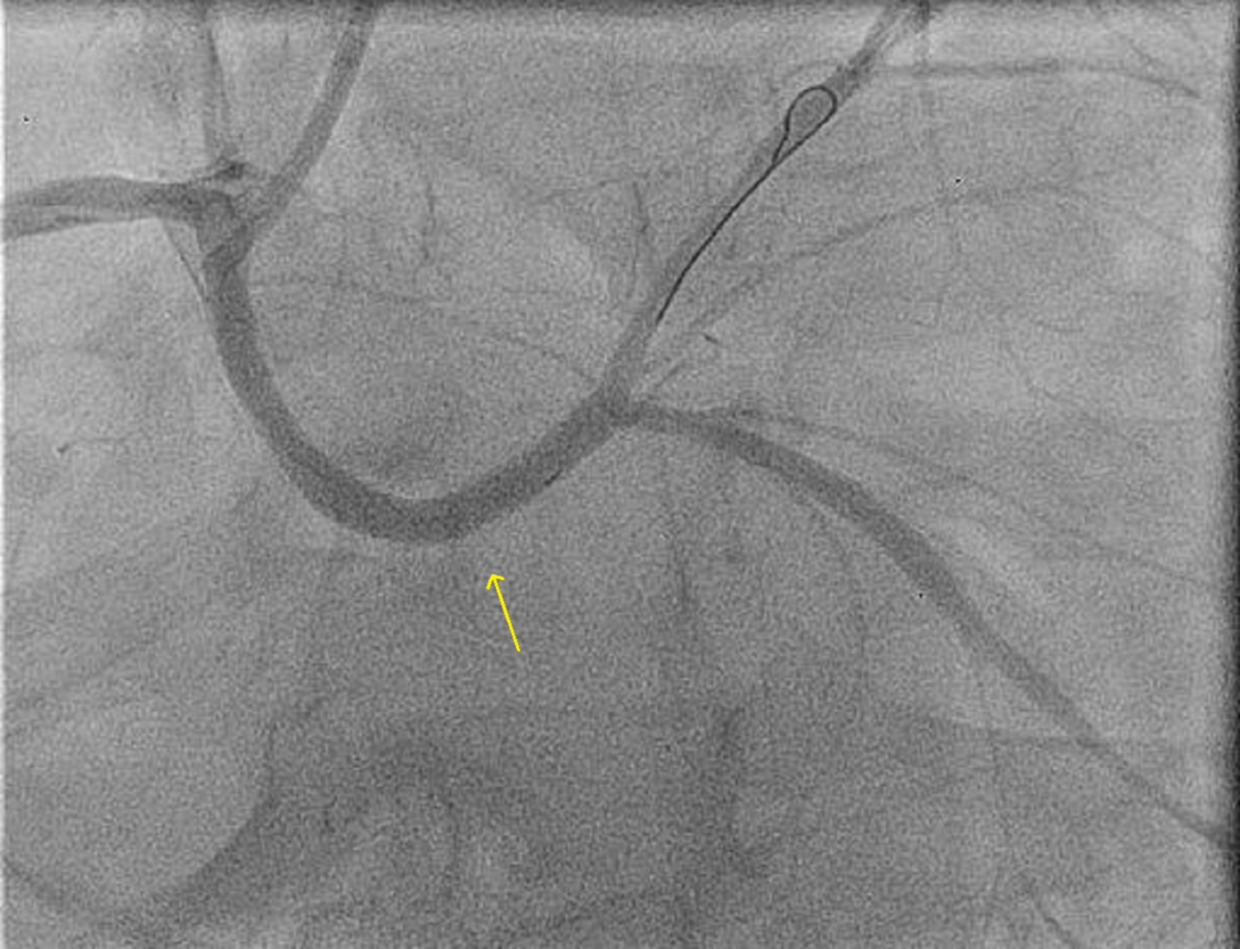
Post in-stent restenosis stenting

Six months later, he was admitted again due to the recurrence of chest pain with a normal ECG and mild troponin rise. The echocardiogram revealed an LVEF of 50%. Of note, the patient quit smoking since discharge. The coronary angiogram revealed significant proliferative type three ISR (10 mm extending beyond the stent margins) (Figure 5). IVUS was done and revealed intimal hyperplasia with no evidence of an obvious stent fracture or malapposition. The patient was offered surgery but he refused. In view of the diffuse pattern of intimal hyperplasia and no stent/native artery size mismatch, we proceeded with the placement of the 4x15 mm Xience stent (Abbott Laboratories, Lake Bluff, Illinois), followed by 4.5x12 mm non-complaint balloon dilation at high pressure (Figure 6). The final IVUS revealed an excellent result.

## Discussion

The clinical incidence of ISR after BMS implantation is approximately 20%-35%. The use of DES has led to a further decrease in the occurrence of ISR to 5%-10% [5]. ISR after coronary angioplasty is currently one of the main limitations of this method, leading to the recurrence of exertional angina pectoris or acute coronary syndromes. Several risk factors contributed to the development of ISR [6]. These include patients, lesions, and procedural factors (Table 1). Angled lesions had a higher incidence rate of ISA over non-angled lesions and were an independent predictor for ISA in several reports. There are several hypotheses behind ISR in angled lesions [6]. First is the mechanical stress (shearing force) from the stent strut to the vessel wall of angled lesions that provokes neointimal hyperplasia. Second, some struts in the inner curvature at the edge would not be well attached to the vessel wall in angled lesions even if the stent was well expanded with recommended balloon pressure. Third, stent recoil was observed in some stent designs. A recent report indicated that stent recoil after the first-generation DES occurred in 0%-2%. Some speculate that stent recoil is prone to occur in hinge motion lesions because excessive vessel movement causes stent compression at the hinge point during the cardiac cycle, and stent recoil causes a decrease in minimal stent area. Fourth, a stent fracture had been reported as the reason for ISR in angled lesions in several studies. Furthermore, small stent fractures, such as a single strut fracture, which is difficult to detect with fluoroscopy or IVUS, might occur at the angled lesions. In one study, they found that the angle of ≥16° is the optimal cut-off value for predicting ISR [2].

**Table 1:** In-stent restenosis risk factors PCI: percutaneous coronary intervention; MLD: minimal lumen diameter

Risk Factors
Patient Factors
Age
Female sex
Genetic factors
Diabetes mellitus
Renal insufficiency
Acute coronary syndrome
Lesion Factors
Long lesions
Small vessels
Complex B2/C lesions
Chronic closures
Ostial lesions
Bifurcation lesions
Lesions in venous bypass
Recurrent restenosis
Multivessel coronary artery disease
Angled lesions
Procedure Factors
Type of stent
Number of stents and total length
Stent overlap
Stent underexpansion
Stent fractures
Post-PCI MLD

The clinical significance of the high incidence of vessel wall injuries and ISA in angled lesions is still unclear. A recent clinical study of second-generation DES reported that lesion angle had no impact on one-year clinical outcomes, inconsistent with a past report in the bare metal stent era [7]. This might be derived from the better mechanical performance of second-generation DES with thinner struts and improved cell design. Some reports have demonstrated that injuries of larger size and ISA would not have healed well, resulting in incomplete healing of vessel injuries and persistent strut malapposition [8]. Therefore, we need further studies to evaluate the long-term clinical outcomes and clinical significance of optical coherence tomography (OCT) findings in patients with angled lesions.

## Conclusions

ISR is not uncommon and several risk factors have been reported as predisposing factors for such unwanted outcomes. Stenting of an angled lesion is associated with a higher incidence of ISR as compared to a non-angled lesion. It has been reported previously in the major coronary arteries. To our knowledge, our case is the first to report such an effect on an anomalous left main coronary artery.
